# MiMiCPy-FM: A User-Friendly Force Matching Tool for Extending the Time Scale of QM/MM MD MiMiC Simulations

**DOI:** 10.1021/acs.jcim.5c03185

**Published:** 2026-02-20

**Authors:** Sachin Shivakumar, Giorgia Frumenzio, Francesco Musiani, Fabio Affinito, Emiliano Ippoliti, Bharath Raghavan, Giulia Rossetti, Davide Mandelli, Paolo Carloni

**Affiliations:** † Computational Biomedicine (INM-9), 28334 Forschungszentrum Jülich, Jülich 52428, Germany; ‡ Department of Physics, RWTH Aachen University, Aachen 52062, Germany; § Department of Applied Physics, Science for Life Laboratory, KTH Royal Institute of Technology, Solna SE-171 21, Sweden; ∥ CINECA, Casalecchio di Reno 40033, Italy; ⊥ Laboratory of Bioinorganic Chemistry, Department of Pharmacy and Biotechnology, 9296 University of Bologna, Bologna 40126, Italy; # National Center for Computational Sciences, Oak Ridge National Laboratory, Oak Ridge, Tennessee 37831, United States; ∇ Jülich Supercomputing Centre (JSC), 28334 Forschungszentrum Jülich, Jülich 52428, Germany; ○ Department of Neurology, University Hospital Aachen (UKA), RWTH Aachen University, Aachen 52062, Germany; ◆ Neuroscience and Neuroimaging (INM-11), 28334 Forschungszentrum Jülich, Jülich 52428, Germany

## Abstract

Force matching (FM) algorithms develop force fields to dramatically extend the time scales of quantum mechanical/molecular mechanics (QM/MM) molecular dynamics (MD) simulations. Here, we present MiMiCPy-FM, an implementation of the generalized QM/MM FM approach for the automated parametrization of biomolecular force fields. MiMiCPy-FM streamlines the optimization of force field parameters by using reference data generated by the recently developed, highly scalable QM/MM MD MiMiC interface. MiMiCPy-FM is fully integrated within the MiMiCPy framework, providing both a command-line interface for quick execution and a Python library for advanced, customizable workflows. The tool is able to treat systems with and without covalent QM/MM boundaries and produces updated topology files that can be directly used to perform classical MD simulations with GROMACS. An application to a complex Mg-based enzyme of pharmacological relevance illustrates how MiMiCPy-FM enables a seamless transition from MiMiC QM/MM MD simulations to long-time scale, force-matched classical MD simulations.

## Graphical abstract

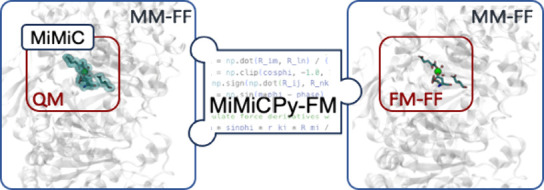

## 1 Introduction

Force matching (FM) approaches derive accurate, system-specific force field parameters directly from higher-level molecular dynamics (MD) reference data.^1–8^ Specifically, the generalized FM approach uses multiscale quantum mechanics/molecular mechanics MD simulations, and it has been developed for applications to biologically relevant systems. This method avoids reliance on gas-phase models or generic parameter sets; instead, it transforms the problem of building a potential energy function into a statistical regression problem based only on the forces. FM ensures that the forces from the force field align with those from the reference QM/MM MD calculation. Provided that the forces are accurate throughout the relevant regions of configuration space, the resulting forces will accurately reproduce the dynamics, which is governed by forces rather than energies.

Several groups, including ours, have applied the method to bulk water and to simple biological systems such as peptides.^9–11^ The implementation of Doemer et al.^2^ in the CPMD-GROMOS QM/MM MD interface^12^ enabled to extend FM to proteins and nucleic acids, where the quantum regions (e.g., the enzymatic active site or the drug and its surroundings) were treated at the first-principles density functional theory (DFT) level. The key limitation of this approach was the poor scalability of the implementation, limiting the time scales that could be reached by QM/MM MD simulations. To address this issue, a group of researchers, including some of us, developed a highly efficient and scalable QM/MM MD interface called Multiscale Modeling in Computational Chemistry (MiMiC).^13–15^ Its current released version, which combines the CPMD^16^ QM package with the GROMACS MD engine,^17–19^ is capable of handling subnanosecond dynamics on modern supercomputers in routine applications.^14,20^ It exploits to an unprecedented extent the capabilities of parallel computing.^20^ The MiMiC QM/MM MD implementation is described in refs 13–15 . In particular, we point the interested reader to our recent detailed overview of the software.^15^ MiMiCPy,^21^ a companion code to MiMiC, simplifies the complex and error-prone task of preparing QM/MM MD input files. Both MiMiC^13,14^ and MiMiCPy^21^ are open source. The online material^22^ provides tutorials and practical guidance for using both MiMiC and MiMiCPy (including MiMiCPy-FM).

Here, we present MiMiCPy-FM, a practical and accessible implementation of the generalized force-matching approach,^2^ a well-established methodology extensively validated and applied by several research groups.^9–11,23,24^ The tool is integrated within MiMiCPy. It provides an automated workflow for deriving Dynamically Generated Restrained Electrostatic Potential (D-RESP) charges^25,26^ and bonded force-field parameters for the atoms in the QM region, starting from QM/MM trajectories obtained using the currently released version of MiMiC,^14,20,21^ which combines the CPMD QM package with the GROMACS MD engine. MiMiCPy-FM automates both the optimization of the force field parameters as well as the generation of updated GROMACS itp files that can be directly used for long-time classical MD simulations. The usability of MiMiCPy-FM is demonstrated by its straightforward pip installation,^27^ single command-line execution, and integration as a Python library. A user manual is also available online.^28^

As with many force field parametrization approaches, the accuracy of the fitting is limited by the short time scales typically accessible in the reference higher-level MD simulations, which restrict phase-space exploration. In this context, our implementation benefits from two main advantages: (i) The highly scalable MiMiC QM/MM MD software is specifically designed to exploit modern massively parallel architectures, enabling the extension of accessible simulation time scales, as demonstrated in our previous works.^14,20^ (ii) Enhanced-sampling techniques, such as metadynamics,^29–31^ are readily available in MiMiC through its interface with state-of-the-art enhanced sampling libraries^15^ that can be used to dramatically accelerate phase space exploration. Furthermore, standard strategies to improve sampling can be straightforwardly applied, such as generating extended conformational ensembles using a lower-level force field—optionally combined with enhanced-sampling schemes (see, e.g., the method of ref 32)—followed by single-point MiMiC-based QM/MM calculations to produce reference data for subsequent force matching.

The ability of MiMiCPy-FM to enhance the accuracy and reliability of multiscale simulations is demonstrated here through its application to a complex biological system such as the Mg­(II)-based enzyme human Isocitrate Dehydrogenase 1 (IDH1).^20,33^

### 1.1 Theory

MiMiCPy-FM implements the FM procedure of Doemer et al.^2^ into MiMiC. It is a fitting procedure that uses, for each QM/MM MD sampled structure (“snapshot” from here on), (i) the total forces ***F*** ^*QM*^ acting on the QM atoms, (ii) the electrostatic potential *V* ^*ρ*^ and electric field ***E*** ^*ρ*^ generated by the QM charge distribution *ρ* at the positions of the short-range (SR) atoms, and (iii) the positions of the QM and SR atoms, namely, the MM atoms within a user-defined cutoff radius from the QM region:^13^

1. Atomic point charges, {*q*_α_} for the QM atoms are fitted using the D-RESP scheme.^2,25,26^ Namely, {*q*_α_} are optimized to reproduce *V* ^*ρ*^ and ***E*** ^*ρ*^, across all the reference snapshots. The fitting is performed by minimizing the following penalty function with respect to {*q*_α_}:
1 $$\chi^{2}\left(\left\{q_{\alpha }\right\}\right)=\overset{L}{\underset{l=1}{\sum }}\underset{\beta \in SR_{l}}{\sum }\left[w^{V}{\left(V_{\beta l}^{\rho }-V_{\beta l}^{MM}\left(\left\{q_{\alpha }\right\}\right)\right)}^{2}+w^{E}{\left|E_{\beta l}^{\rho }-E_{\beta l}^{MM}\left(\left\{q_{\alpha }\right\}\right)\right|}^{2}\right]+w^{H}\underset{\alpha }{\sum }\left(q_{\alpha }-q_{\alpha }^{H}\right)+w^{Q}{\left(\underset{\alpha }{\sum }q_{\alpha }-Q^{tot}\right)}^{2}$$
  where *L* is the number of snapshots; β runs through the set of SR_*l*_ atoms in the *l*th snapshot; *V* *_βl_^ρ^* and **E** *_βl_^ρ^* are the QM electrostatic potential and field at SR atom β in snapshot *l*; *V* *_βl_^MM^* and **E** *_βl_^MM^* are the corresponding quantities computed using the set of fitted point charges; *q*_α_^H^ is the restraint charge for the QM atom α (typically the reference charge from the original force field or the Hirshfeld charge^34^ obtained during the QM/MM reference calculation); *Q* ^*tot*^ is the total charge of the QM region; *w* ^*V*^, *w* *^E^*, *w* *^H^*, *w* *^Q^* are weighting factors for the potential, field, restraint charges, and total charge constraint terms, respectively. The four weights control the relative importance of the four terms that ensure: (i) reproducing the QM/MM electrostatic potential, (ii) reproducing the QM/MM electric field, (iii) enforcing total charge consistency, and (iv) regularizing the fitted partial charges toward physically reasonable values. These weights allow flexibility to balance accuracy and physicality in the fitting procedure. We note that total charge conservation is strictly enforced, and an error is raised if it is not achieved during the fitting procedure. The minimization of χ^2^ is usually recast as a linear least-squares problem.^2^
2. Nonbonded electrostatic and dispersion forces acting on the QM atoms are computed using the newly fitted D-RESP charges and the same van der Waals parametrization used in the reference MiMiC QM/MM MD simulation, respectively. Their sum, ***F*** ^*MM,nb*^, is subtracted from the total QM/MM reference forces, ***F*** ^QM^, isolating the “bonded part” of the reference forces. This, in turn, is fitted using classical bonded terms, ***F*** ^*MM,bonded*^ {*τ*_*n*_}. In short, the set {*τ*_*n*_} of bonded parameters of the QM region are obtained by minimizing the objective function:
2 $$\sigma^{2}\left(\left\{\tau_{n}\right\}\right)=\overset{L}{\underset{l=1}{\sum }}\underset{\alpha \in QM}{\sum }{\left|F_{l\alpha }^{QM}-F_{l\alpha }^{MM,\mathrm{nb}}-F_{l\alpha }^{MM,\mathrm{bonded}}\left(\left\{\tau_{n}\right\}\right)\right|}^{2}$$
  where **F**_*lα*_^*QM*^ are the reference QM/MM forces, **F**_*lα*_^*MM,nb*^ are the nonbonded forces (electrostatic plus van der Waals) computed with the D-RESP charges obtained at step 1, and **F**_*lα*_^*MM,bonded*^ ({τ_*n*_)} are the bonded forces computed using the new set of bonded parameters.

Our implementation, discussed below, provides flexible control over both the parametrization of the objective functions and the minimization strategy, enabling users to adjust weighting factors, select specific terms, or sequence minimization steps as desired.

## 2 Implementation of the FM Module

MiMiCPy-FM handles tasks such as data management, parameter optimization for electrostatic and bonded terms, and topology files updating. Its implementation follows a modular design that spans (i) the MiMiC^15^ library, version 0.2.0 (which couples CPMD and GROMACS), and (ii) MiMiCPy.^21^ Modifications to store and output the reference forces and electrostatic data during QM/MM MD simulations are implemented in the first, while the data management and optimization processes are implemented in the second.

### 2.1 Modification of MiMiC

We have modified the mimic_short_range module and added a new mimic_force_matching module to MiMiC. The new module (i) stores the forces on the QM atoms and the electrostatic potential and field at the positions of the SR atoms for each snapshot and (ii) outputs this data in JSON format to the FMTRAJECTORY.json output file, with per-snapshot data stored as a separate JSON object. These modifications automate the collection of all the reference data directly during MiMiC simulations, eliminating the need for additional postprocessing steps.

### 2.2 Modification of MiMiCPy

#### 2.2.1 I/O and Data Management

##### 2.2.1.1 Topology Files Handling

This has been significantly enhanced through comprehensive updates to the existing top and itp classes. These classes now provide sophisticated parsing and modification capabilities for GROMACS topology files, supporting both reading and writing of complex molecular structures. Extensions include robust handling of “include” directives, molecule definitions, and parameter sections.

##### 2.2.1.2 Reference Data Management

The snapshots are managed by the new FMData set class. Methods such as get_configuration_properties provide streamlined access to specific data slices while maintaining memory efficiency even for data sets containing thousands of configurations. The class handles both the JSON and HDF5 formats. The HDF5 backend enables the processing of large-scale data sets that would be otherwise impractical using JSON.[Fn fn1]

##### 2.2.1.3 QM Region Management

The QMRegion class manages all the QM region-related information during FM: it handles the selection of the QM atoms through flexible selection strings, following the syntax rules of the prepqm class;^21^ it holds the atomic index mappings between different codes (GROMACS, internal, and CPMD indexing); and it provides functionalities for extracting force field parameters related to the QM region from the GROMACS topology files. Key features include equivalent atom mapping for maintaining chemical symmetry and automatic determination of the bonded parameters to be fitted. The class also handles topology updates using methods such as update_topology and write_topology, generating appropriately prefixed output files (e.g., opt_, resp_, non_bonded_) with optimized parameters.

##### 2.2.1.4 User Input

The FMInput interface controls the FM procedure through a keyword-based system. It supports both basic usage with sensible defaults and advanced scenarios with detailed parameter specification (see SI). All parameters can be provided through a dedicated FM input file, when FM is performed from the command line (see the “-fi” option described in Section 2.3) or via dedicated methods, when MiMiCPy is used as a Python library for advanced scripting. The class also implements error checking and validation of the user-provided options to ensure parameter consistency.

#### 2.2.2 Optimization Components

##### 2.2.2.1 D-RESP fitting

The dresp.py module implements the D-RESP fitting procedure.^2,25,26^ The optimization is performed by the opt_dresp function, which constructs the penalty function of eq 1 by using user-specified weighting factors. Specifically, for each snapshot, the electrostatic potential and electrostatic field terms of eq 1 are computed analytically in the compute_potential_set_charges and compute_electric_field_set_charges functions, respectively. These terms can be computed straightforwardly when no covalent bonds cross the QM–MM boundary. This cannot be done in systems with boundary atoms^2^ and, as in ref.,^2^ we adopted a charge redistribution scheme where, first, the D-RESP procedure is applied straightforwardly, then, the charges on a user-defined number of neighboring QM atoms from the boundary atom are set to their original force field values, and, last, any ensuing excess charge is redistributed across the QM region to ensure conservation of the total charge.

In practice, the least-squares problem is solved using NumPy’s linear least-squares solver (np.linalg.lstsq). Throughout the optimization process, chemical symmetry is maintained by ensuring that equivalent atoms receive identical charges. The total charge is strictly conserved, and an error is raised if it is violated.

Different combinations of weighting factors (*w* ^*V*^, *w* ^*E*^, *w* ^*H*^, and *w* ^*Q*^) can be explored automatically: the factors can be optionally supplied by the user as a parameter grid rather than single values. This is particularly useful to systematically explore parameter space and find the optimal solution to the minimization problem. When the grid option is used, the module automatically identifies the best set of parameters as the one that minimizes the sum of the mean square deviation between the reference electrostatic quantities and those computed using the final optimized point charges. All the results are also exported to a CSV file to allow detailed inspection.

The dresp.py module implements parallel processing capabilities through the simultaneous processing of multiple snapshots in functions such as _compute_sd_parallel and _compute_influence_mat_parallel using the Python multiprocessing module. This is expected to offer nearly linear scaling for the computation of the D-RESP objective function, substantially improving the performance, particularly for large systems.

##### 2.2.2.2 Bonded Parameter Optimization

**Analytical Force Computation**: The bonded_forces.py module implements the analytical forms of all bonded force terms contributing to **F** ^*MM,bonded*^({τ_*n*_)} in eq 2), ensuring compatibility with their GROMACS implementation (see SI).
**Reference Bonded Force Extraction**: The reference bonded forces are defined in eq 2) by the difference **F** ^*QM*^ – **F** ^*MM,nb*^ between the total QM/MM forces and the MM nonbonded forces computed using the fitted D-RESP charges. The latter are computed using GROMACS by preparing a topology file in which all bonded force constants involving QM atoms are set to zero, therefore isolating the nonbonded interactions only and performing a rerun against the reference trajectory.
  The rerun uses the same GROMACS.mdp file that was employed during the QM/MM simulations, with the requirement that the `nstfout̀ keyword must be set to 1 to output forces. The GROMACS executable and the mdp file are specified via command-line options (see section 2.3 for details). Our implementation fully automatizes the topology modification, rerunning GROMACS, and extraction of the forces **F** ^*MM,nb*^. For efficient computation, GROMACS can be executed by OpenMP parallelization. The number of threads can be controlled via the relevant environment variable (e.g., the OMP_NUM_THREADS).
**Optimization Strategies**: The objective function of eq 2) is minimized using Scipy’s least_squares optimizer. Our implementation provides (i) options for either hierarchical, simultaneous or user-defined strategies, (ii) techniques to mitigate overfitting, and (iii) built-in parallelization features.
  (i) The hierarchical optimization strategy recognizes the natural energy scale separation between different interaction types, optimizing parameters in stages: first bonds, then angles, and finally dihedrals. This sequential approach prevents lower-energy terms from affecting the accuracy of the fitting of higher-energy interactions. The simultaneous optimization strategy optimizes all parameters concurrently, which can be more efficient, but careful weighing of the different contributions is required. Beyond these two main optimization strategies, the user retains full control over which terms (bonds, angles, dihedrals) are included or excluded from the optimization. An important consideration for dihedral parameters is that QM/MM simulations are typically not long enough to adequately sample all accessible dihedral conformations. Fitting dihedral parameters to such incomplete conformational sampling can lead to unstable parameters that may not generalize to conformations not present in the reference data. Therefore, dihedral parameters are often kept at their original force field values unless sufficient conformational sampling is available.
  (ii) To prevent overfitting and ensure the parameters remain physically reasonable, we have implemented the option to include the L2 regularization term of ref 35.
  (iii) The implementation employs parallel processing to efficiently compute forces across multiple snapshots simultaneously, distributing the workload among available CPU cores.

### 2.3 Usage and Workflow

Figure 1 illustrates the complete MiMiCPy-FM workflow. Initially, reference data are generated during a QM/MM MD MiMiC simulation. To enable FM data collection in the FMTRAJECTORY.json output file, the FORCE_MATCHING keyword must be specified in the CPMD input file. Subsequently, FM is performed with MiMicPy. The MiMiCPy FM module provides a command-line interface via the mimicpy fm command, which can be invoked as follows:
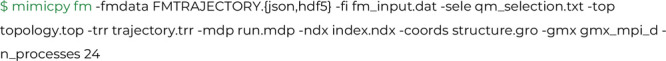


**Figure 1:**
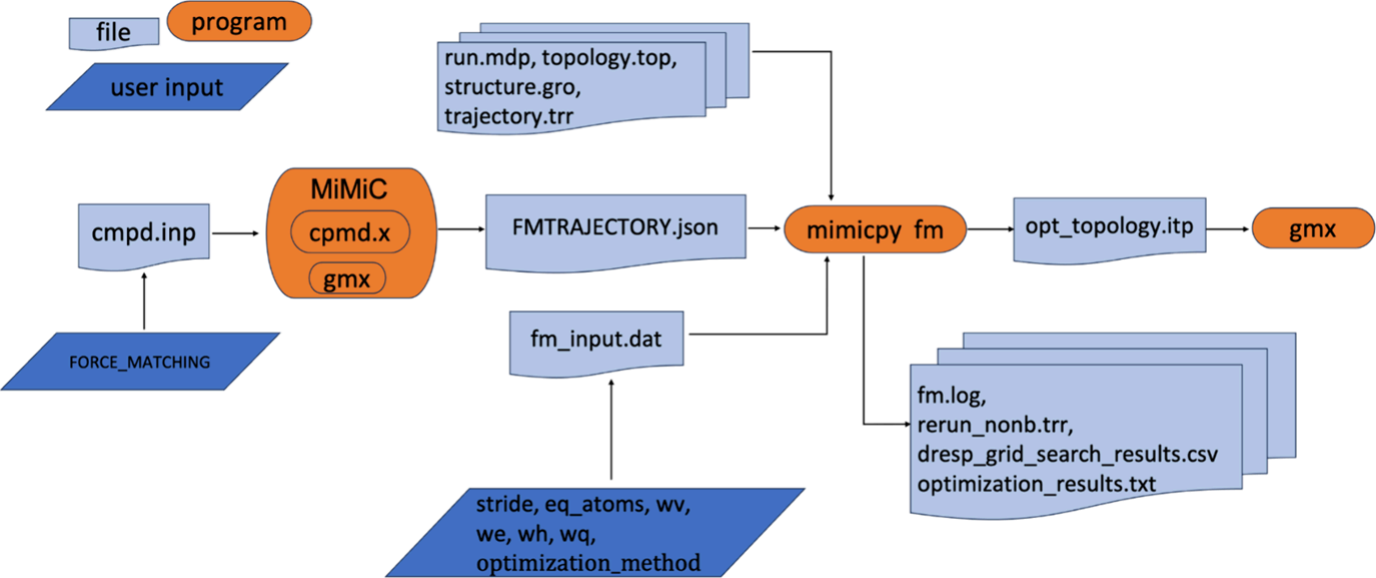
Flowchart for FM performing using MiMiCPy-FM.

This activates a workflow through the fm function. Several input files are loaded using dedicated keywords, these include:

- the reference data in JSON or HDF5 formats (-fmdata)
- the FM input parameters (-fi, see SI for the complete list of options)
- the QM atoms selection (-sele)
- the topology file with the original force field (-top)
- the GROMACS trajectory generated during the reference MiMiC QM/MM MD simulation (-trr)
- a GROMACS simulation parameter file (-mdp)
- a GROMACS index file (-ndx)
- a GROMACS coordinate file (-coords)
- the GROMACS executable (-gmx) needed for the rerun procedure
- the number of processes to use for parallel execution (-n_processes).

The workflow proceeds with D-RESP charge optimization. The GROMACS rerun is then performed to extract the nonbonded forces. Next, the bonded force field optimization is performed. The entire process is parallelized based on the specified number of processes.

The output files include:

- the fm.log file that tracks the various steps of the workflow in real time
- the rerun_nonb.trr file generated by the GROMACS rerun
- the optimization_results.txt file containing the results of the least-squares minimization of the objective function of eq 2)
- the optimized topology file opt_topology.itp containing the QM atoms and their force matched parameters
- If the grid search option is used for the weighting factors of eq 1, the dresp_grid_search_results.csv file is also generated, containing the results of the D-RESP optimization at each grid point.

Finally, the optimized topology file can be used to run classical MD simulations using GROMACS.

The optimal choice of FM parameters is generally system-dependent. The strategies used here for the two case studies provide a practical reference for setting up the fitting procedure in other systems, as well. For all systems studied here, the fitting procedure was robust, and no instabilities or convergence issues were observed during the optimization of the partial charges and bonded parameters.

## 3 Validation: Acetone in Water

The validation of the MiMiCPy-FM approach was performed by applying it to QM/MM MD simulations of an acetone molecule (QM region) in explicit water (MM region). We assessed the quality of the fit by computing the normalized standard deviations of the fitted electrostatic potential (σ_V_, eq S5), electrostatic field (σ_E_, eq S6), and forces (σ_F_, eq S6), from the reference QM/MM values. The final values of σ_V_ = 0.11, σ_E_ = 0.34, and σ_F_ = 0.65 are in line with those of typical FM fitting using the same method,^1,2^ indicating that the optimization was successful, validating our implementation. Comparing the FM parameters with those of the original GAFF parametrization (Tables S1–S4), the main observation is a slight increase in the partial charge of the carbonyl oxygen (Table S1). Additionally, we compared the accuracy of the FM potential relative to the reference QM/MM MD data. Compared to the original GAFF parametrization, results show that the FM parametrization better reproduces the internal structural dynamics of the acetone molecule (Figures S2–S4), but leads to a somewhat less accurate prediction of the solvation structure (Figure S5).

The online material provided in the associated Zenodo repository includes scripts and output files used for the fitting, providing a step-by-step tutorial for the utilization of the MiMiCPy-FM tool.

## 4 Application: Isocitrate Dehydrogenase 1 (IDH1)

Isocitrate dehydrogenase 1 (IDH1) is a dimeric, magnesium-based enzyme catalyzing the oxidative decarboxylation of isocitrate to 2-oxoglutarate (αKG).^36–38^ Its R132H mutant converts αKG to the oncometabolite 2-hydroxyglutarate^39^ and is an important target for therapies against brain cancers.^33^ The structural determinants of the Michaelis complex of this mutant, predicted by some of us by 20 ps long MiMiC calculations,^33^ contains the Mg^2+^ ion, the αKG substrate, and the NADPH cofactor required for the enzymatic reaction (Figure 2). The QM region in the MiMiC calculations included the substrate, the metal coordination polyhedron, part of the cofactor, and additional residues indicated in Figure 2.

**Figure 2:**
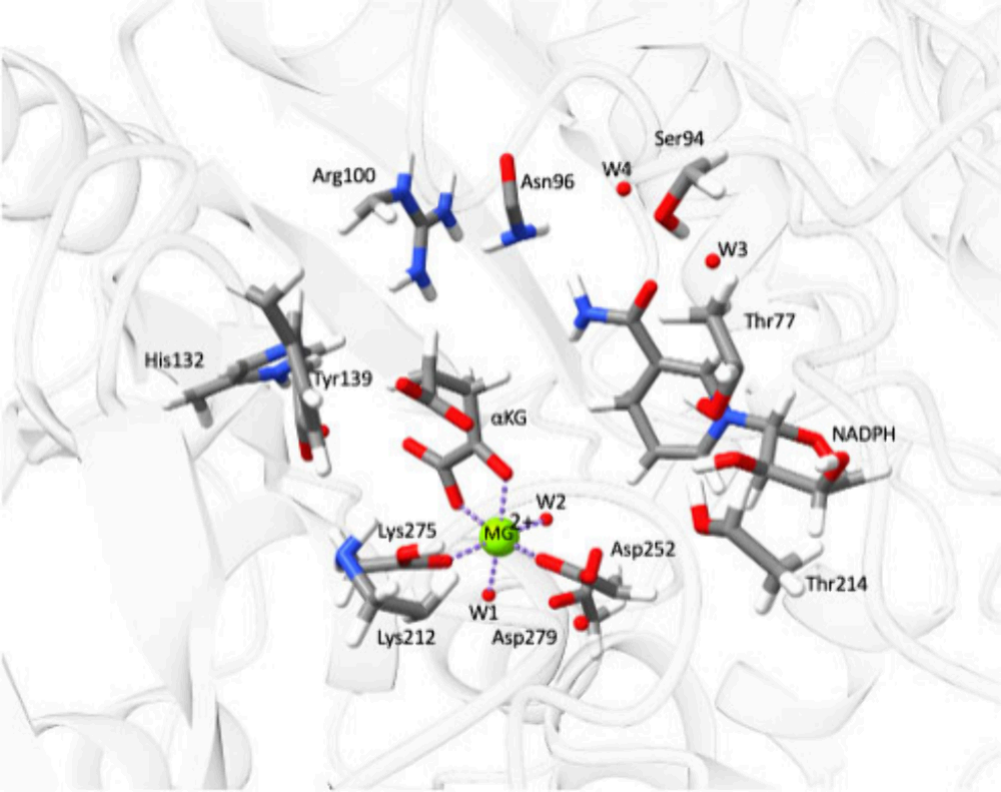
Detail of the R132H IDH1 active site as simulated in QM/MM MD^33^ showing the Mg^2+^ coordination polyhedron, the αKG substrate, and the NADPH cofactor. Protein ribbons are in shaded gray, while QM atoms are colored according to their element type: C: dark gray, N: blue, H: white, O: red, Mg: green.

The FM procedure included all QM atoms except the water molecules in order to ensure a consistent solvent description. That is, water parameters were not fitted in the QM region so that the same TIP3P model was used for all solvent molecules, including during the fitting stage. A total of 1053 reference configurations were extracted from the QM/MM MD trajectory. Atomic point charges for the QM atoms were calculated using the DRESP methodology with weights w_v_ = 1, w_e_ = 0.1, w_h_ = 100, and w_q_ = 100,000. Bonded parameters were optimized by using the hierarchical optimization strategy. Dihedral parameters were kept at their original force field values since the QM/MM simulation time scales are insufficient to sample all accessible conformations (see SI for details). The final values of σ_V_ = 0.98 and σ_E_ = 0.99 show that the electrostatic potential and field are reasonably reproduced by the parametrization over the training set, while larger deviations are observed for the forces (σ_F_ = 33.0). This discrepancy may be partly attributed to the choice in the FM procedure to treat water molecules in the QM region using fixed TIP3P parameters rather than including them explicitly in the fitting. Importantly, despite these deviations at the force level, MD simulations (discussed below) show that the parametrization robustly preserves the reference QM-region structure over microsecond time scales.

Classical MD simulations used the FM-based force field for the QM part and exactly the same MM force field as that used in the reference QM/MM calculations.^33^ They were carried out with GROMACS.^17^ The equations of motion were integrated with a time step of 2 fs, and all bonds involving hydrogen atoms were constrained by using the LINCS algorithm. Temperature was controlled using the velocity-rescaling thermostat, while pressure was maintained at 1 bar with the Parrinello–Rahman barostat. Long-range electrostatic interactions were treated with the Particle Mesh Ewald (PME) method using a real-space cutoff of 1.1 nm, while van der Waals interactions were truncated at 1.0 nm and corrected for long-range dispersion effects on energy and pressure. The last frame from the QM/MM MD trajectory was used to perform a first energy minimization. Then, the system was heated to 300 K under the NPT conditions. The last snapshot was used to run three independent replicas (R#1–3) of 1 μs each, starting from different velocities extracted from a Maxwell–Boltzmann distribution at 300 K.

The simulations reproduced the overall structure of the protein (Figure 3). The T-PAD analysis^40^ (Figure 3b) reveals that the largest part of the residues in the three simulations are fluctuating around a single main geometry, without experiencing large conformational transitions. The expected^33^ octahedral coordination geometry of the Mg^2+^ ion is well maintained (Figure 3c): in all the replicas, the metal ion is coordinated by two aspartate residues (Asp252 and Asp275′). In R1, Asp252 is involved in a bidentate coordination with the metal ion. A different number of water molecules (1 in R#1, 2 in R#2, and 3 R#3) complete the octahedral Mg^2+^ polyhedron. The same coordination number was observed in the QM/MM MD simulations of ref 33. The rest of the active site shows few differences relative to the QM/MM structures (Figure S6). The αKG coordination is different with respect to the starting one and is found to be bidentate (R#1 and R#2) or monodentate (R#3). We may expect these changes as we extend the QM/MM MD time scale (subns)^33^ to that of classical MD (μs).

**Figure 3:**
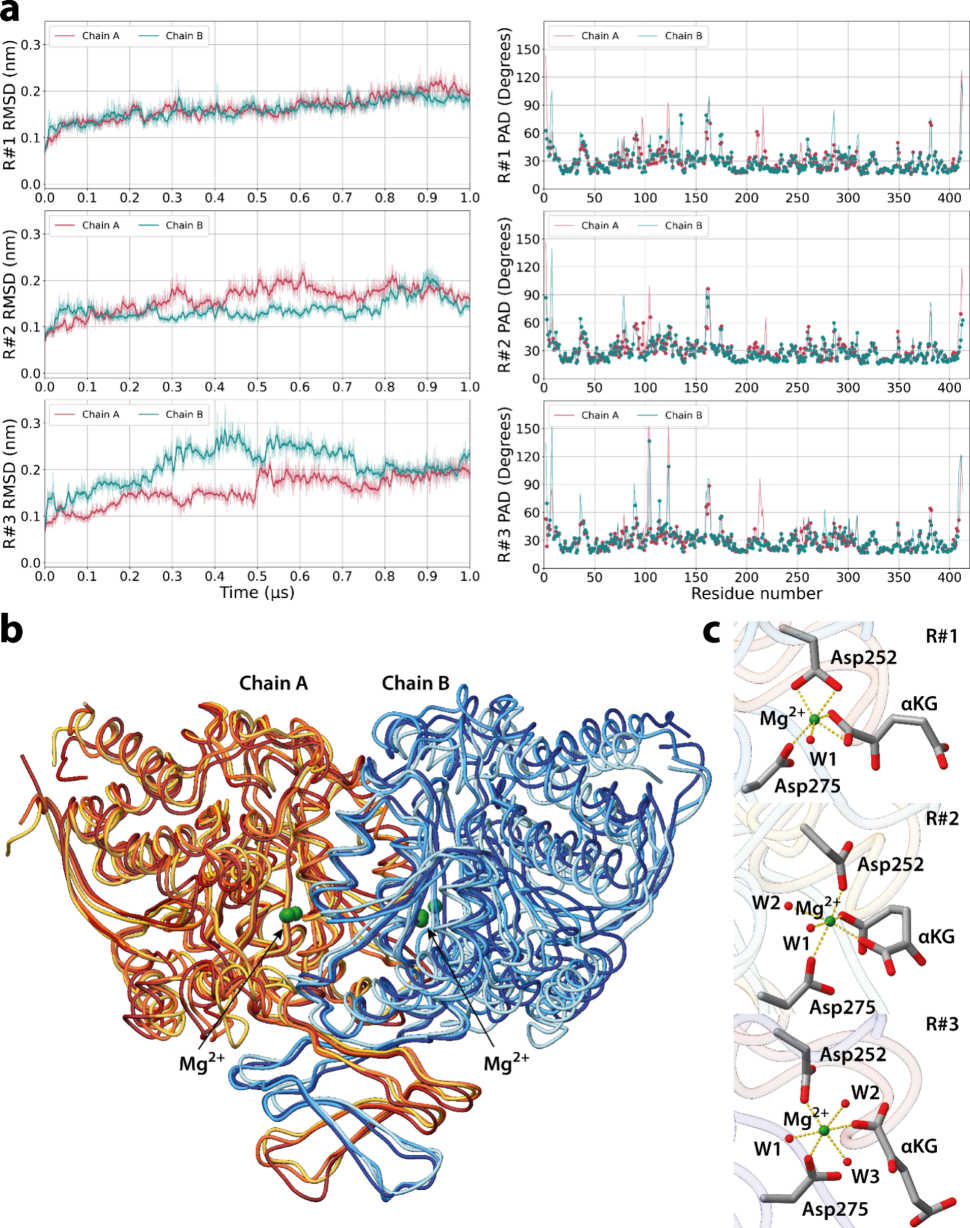
Microsecond long MD simulations of R132H IDH1, based on FM-potentials from QM/MM MD simulations of ref 33. The protein is a dimer, made by chains A and B shown here. The FM procedure was performed in chain B. (a) RMSD (left) plotted as a function of simulated time and T-PAD (right) plotted for each residue for the three replicas (R#1–3). Red and green curves are results for chains A and B, respectively. (b) Overlap between the ribbon representation of the last frame of the three replicas. Moving from R#1 to R#3, the last frames of the three replicas are colored from red to yellow for chain A and from blue to light blue for chain B. The metal ions are represented as green spheres. (c) Mg^2+^ ion coordination polyhedron in the last frame of each replica (R#1–3).

## 5 Conclusions

We have presented MiMiCPy-FM, a tool that automates force field generation from QM/MM MD trajectories generated via the MiMiC interface for QM/MM MD. MiMiCPy-FM combines D-RESP-fitted electrostatics and force-matched bonded parameters, handles QM regions with or without covalent boundary crossings, and outputs GROMACS-compatible itp files, enabling seamless transition to long-time scale classical MD simulations. The combination of a command-line interface and a Python library ensures flexibility for both rapid deployment and advanced custom workflows. The application of the tool to a pharmacologically relevant Mg-based enzyme demonstrates its capability to maintain fidelity to QM/MM MD simulations.

## Supplementary Material



## Data Availability

MiMiCPy is pip-installable via “pip install mimicpy” (https://pubs.acs.org/doi/10.1021/jp044629q). The source is available on GitLab at http://gitlab.com/mimic-project/mimicpy, published under the GNU Lesser General Public License version 3 or later (LGPLv3+). The user manual of MiMiCPy-FM can be found at https://mimic-project.org/en/latest/mimicpy/force_matching.html. Additional information about software needed for running MiMiC-based QM/MM simulations is available at https://mimic-project.org. The Zenodo repository (http://doi.org/10.5281/zenodo.17975944) contains input data and scripts used to perform FM of the systems studied here.

## Footnotes

a In a typical force-matching workflow, users often perform multiple minimizations to test different strategies. This would require repeatedly parsing the reference data contained in the FMTRAJECTORY.json file. Reading JSON files can be highly time-consuming for large systems and/or long trajectories. To mitigate this overhead, the reference data can be exported to an HDF5 file during the first minimization, allowing all subsequent runs to load the compressed HDF5 data directly for substantially faster access.

## Notes

A full list of available input parameters for force matching, further details on the analytical expressions implemented for the computation of bonded forces, results and discussion of the application of MiMiCPy-FM to acetone in water and IDH1 (PDF)
