# Supplementary material for: MiMiCPy-FM: A User-Friendly Force Matching Tool for Extending the Time Scale of QM/MM MD MiMiC Simulations
Source: J Chem Inf Model. 2026 Feb 20;66(5):2458–65. doi: 10.1021/acs.jcim.5c03185 (PMC12977040; doi:10.1021/acs.jcim.5c03185)
Supplement: Supplementary file 1 [file ci5c03185_si_001.pdf]

Supporting Information

MiMiCPy-FM: a user-friendly force matching tool for extending the time scale of QM/MM MD MiMiC simulations

Authors:
Sachin Shivakumar[†1,2,3], Giorgia Frumenzio[†4,5], Francesco Musiani[5], Fabio Affinito[4], Emiliano Ippoliti[1], Bharath Raghavan[6], Giulia Rossetti[1,7,8], Davide Mandelli[*1], and Paolo Carloni[*1,9]

Affiliations:
[1] Computational Biomedicine (INM-9), Forschungszentrum Jülich, Jülich, Wilhelm-Johnen-Straße, 52428, Jülich, Germany
[2] Department of Physics, RWTH Aachen University, 52062 Aachen, 52062 , Germany
[3] Department of Applied Physics, Science for Life Laboratory, KTH Royal Institute of Technology, SE-171 21 Solna, SE-171 21, Sweden
[4] CINECA, Casalecchio di Reno, 40033, Bologna, Italy
[5] Laboratory of Bioinorganic Chemistry, Department of Pharmacy and Biotechnology, University of Bologna, Bologna, 40126 , Italy
[6] National Center for Computational Sciences, Oak Ridge National Laboratory, Oak Ridge, Tennessee, 37831, USAnited States of America
[7] Jülich Supercomputing Centre (JSC), Forschungszentrum Jülich, Wilhelm-Johnen-Straße, 52428, Jülich, 52428, Germany
[8] Department of Neurology, University Hospital Aachen (UKA), RWTH Aachen University, Aachen, 52062, Germany
[9] Neuroscience and Neuroimaging (INM-11), Forschungszentrum Jülich, Wilhelm-Johnen-Straße, 52428, Jülich, 52428, Germany

† Shared first authorship
* Corresponding author

1. List of Available Input Parameters

Below, we provide the full list of parameters in the force matching input file, including default values for optional parameters that need initialization. For ease of reference, we organize them in different categories.

Trajectory sub-sampling

- stride: List of three integers [start, end, step]. Mandatory. It defines the starting snapshot, the final snapshot, and the sampling frequency of the reference data to be used for the fitting.

Chemical Equivalence Definitions

- eq_atoms: Mandatory. Accepted values:

- 'use_atomtypes', automatically determines equivalence from atom types

- 'eq_atoms_global', followed by a list of tuples containing the (gromacs) indices of equivalent atoms
- 'eq_atoms_local', followed by a dictionary containing molecule names and the list of equivalent atoms in the molecule using

D-RESP Fitting Parameters

- wv: Float or list of floats. Mandatory. Weight for electrostatic potential fitting. It can be a single value or list for grid search.

- we: Float or list of floats. Mandatory. Weight for electric field fitting. It can be a single value or list for grid search.

- wh: Float or list of floats. Mandatory. Weight for Hirshfeld charge restraints. It can be a single value or list for grid search.

- wq: Float. Optional. Weight for total charge constraint (default: 100,000).

- reference_charges: String of list of floats. The string 'ff_charges' instructs the code to use the original force field charges. Alternatively, the user can provide a list of floats to manually set the reference charges of the QM atoms following the internal mimicpy indices for the QM atoms. The internal indices can be printed by performing a preliminary dry run of mimicpy, using the following command: $mimicpy qminfo

- qm_total_charge: Float. Mandatory. Total charge constraint for QM region.

- num_bonds_away: Integer. Optional. Number of bonds away from boundary to include in charge redistribution (default: 2).

- charge_group_constraints: Boolean. Optional. Enforce charge group constraints (default: True).

- fixed_charge_indices: List of integers. Optional. Atom indices with fixed charges (defaults: no fixed charges).

- weights_to_fix_charges: Float. Optional. Weight to fix charges to original values (default: 100000.0).

Solvent Handling

- skip_solvent_optimization: Boolean. Optional. Skip optimization of solvent molecules (default: True).

- solvent_resnames: List of strings. Optional. Residue names to treat as solvent.

- solvent_molecules: List of strings. Optional. Residue names to treat as solvent.

Bonded Parameter Optimization Controls

- optimize_bond_length: Boolean. Optional. Optimize equilibrium bond lengths (default: True).

- optimize_bond_force:Boolean. Optional. Optimize bond force constants (default: True).

- optimize_angle_value: Boolean. Optional. Optimize equilibrium angle values (default: True).

- optimize_angle_force: Boolean. Optional. Optimize angle force constants (default: True).

- optimize_dihedral_force: Boolean. Optional. Optimize dihedral force constants (default: True).

Excluded Interactions

- exclude_bonds: List of tuples. Optional. Atom pairs [(atom1, atom2), ...] to exclude from fitting.

- exclude_angles: List of tuples. Optional. Atom triplets [(atom1, atom2, atom3), ...] to exclude from fitting.

- exclude_dihedrals: List of tuples. Optional. Atom quartets [(atom1, atom2, atom3, atom4), ...] to exclude from fitting.

- exclude_hydrogen_bonds: Boolean. Optional. Exclude all bonds involving hydrogen (default: False).

- exclude_hydrogen_angles: Boolean. Optional. Exclude all angles involving hydrogen (default: False).

- exclude_hydrogen_dihedrals: Boolean. Optional. Exclude all dihedrals involving hydrogen (default: False).

Regularization Settings

- regularization: Boolean. Optional. Enable L2 regularization (default: True).

- regularization_alpha: Float. Optional. L2 regularization strength α (default: 0.1).

Optimization Method Selection

- optimization_method: String. Optional. Accepted values: 'hierarchical' or 'simultaneous'. It selects the strategy to be used for the optimization of the bonded parameters (default: 'hierarchical').

Minimal example of an input file

```
stride: [50,1103,1]
wv: 1
we: 0.1
wh: 100
wq: 100000
eq_atoms: use_atomtypes
optimize_dihedral_force: False
reference_charges: ff_charges
optimization_method: hierarchical
```

2. Analytical Bonded Force Computations

We have implemented analytical expressions for the bonded forces and their derivatives with respect to the fitting parameters. Below, we provide the expressions for the bond, angle and dihedral

potential energy functions we have considered. We have thoroughly validated our implementation by comparing the values of the forces as computed in our MiMiCPy implementation with those obtained directly from GROMACS. The current implementation is limited to the force fields listed below; however, interested users can readily extend it by implementing additional expressions.

Bonds (gromacs bond function type 1):

$$V(r_{ij}) = (K_{ij}/2)(r_{ij}-R_{0,ij})^2 \quad (S1)$$

where r_{ij} is the distance between atoms *i* and *j*. K_{ij} and $R_{0,ij}$ are the corresponding spring constant and equilibrium length to be fitted.

Angles (gromacs angle function type 1)

$$V(\theta_{ijk}) = (K_{ijk}/2)(\theta_{ijk}-\theta_{0,ijk})^2 \quad (S2)$$

where θ_{ijk} is the angle between atoms *i, j,* and *k*. K_{ijk} and $\theta_{0,ijk}$ are the corresponding spring constant and equilibrium values to be fitted.

Dihedrals

For the dihedrals, we implemented two different expressions:

1) Ryckaert-Bellemans (gromacs dihedral function type 3):

$$V(\varphi_{ijkl}) = C_{0,ijkl} + C_{1,ijkl}\cos(\varphi_{ijkl}-180°) + C_{2,ijkl}\cos^2(\varphi_{ijkl}-180°) + C_{3,ijkl}\cos^3(\varphi_{ijkl}-180°) + C_{4,ijkl}\cos^4(\varphi_{ijkl}-180°) + C_{5,ijkl}\cos^5(\varphi_{ijkl}-180°) \quad (S3)$$

where φ_{ijkl} is the dihedral angle defined by atoms *i*, *j*, *k*, and *l.* $C_{0,ijkl}$ through $C_{5,ijkl}$ are the corresponding Ryckaert-Bellemans coefficients to be fitted.

2) Periodic (gromacs dihedral function types 1, 4, 9):

$$V(\varphi_{ijkl}) = K_{ijkl}[1 + \cos(n\varphi_{ijkl} - \varphi_{0,ijkl})] \quad (S4)$$

where φ_{ijkl} is the dihedral angle defined by atoms *i*, *j*, *k*, and *l*. K_{ijkl}, $\varphi_{0,ijk}$ are the corresponding force constant and equilibrium values to be fitted. We note that the multiplicity *n* is not used as a fitting parameter here, instead its value is kept equal to its original GAFF value.

3. Assessing the D-RESP and force fitting results

To assess the quality of the D-RESP and force fitting, the following three indicators are computed:

$$\sigma_V = \sqrt{\frac{\sum_{l=1}^{L}\sum_{\beta\in SR_l}\left(V_{\beta l}^{\rho} - V_{\beta l}^{MM}\right)^2}{\sum_{l=1}^{L}\sum_{\beta\in SR_l}\left(V_{\beta l}^{\rho}\right)^2}} \quad (S5)$$

$$\sigma_E = \sqrt{\frac{\sum_{l=1}^{L}\sum_{\beta\in SR_l}\left|\mathbf{E}_{\beta l}^{\rho} - \mathbf{E}_{\beta l}^{MM}\right|^2}{\sum_{l=1}^{L}\sum_{\beta\in SR_l}\left|\mathbf{E}_{\beta l}^{\rho}\right|^2}} \quad \text{(S6)}$$

$$\sigma_F = \sqrt{\frac{\sum_{l=1}^{L}\sum_{\beta\in QM}\left|\mathbf{F}_{\beta l}^{\rho} - \mathbf{F}_{\beta l}^{MM}\right|^2}{\sum_{l=1}^{L}\sum_{\beta\in QM}\left|\mathbf{F}_{\beta l}^{\rho}\right|^2}} \quad \text{(S7)}$$

$\boldsymbol{\sigma}_{V,E,F}$ respectively measure the deviation of the fitted electrostatic potential, electrostatic field and forces from the reference values, across the training set, following ref[1].
The three standard deviations are reported in the `fm.log` file generated by mimicpy, labeled as $SD_{V,E,F}$.

4. Acetone in water

QM/MM simulations

The setup and protocol used to generate the reference QM/MM data of acetone in water is described in detail in the online MiMiC acetone tutorial (mimic-project.org/en/latest/tutorials/acetone_tutorial.html). Specifically, we ran 24 ps of production NVT QM/MM MD at T=300 K, from which we extracted 2,000 snapshots (one every 50 steps). Last 1000 snapshots were used for the force matching.
For reproducibility, all the input files used to generate the reference data can be found in the Zenodo repository associated with this paper (https://doi.org/10.5281/zenodo.17975944).

Force matching

The FM parameters have been generated using the following MiMiCPy command:

```
$mimicpy fm -top acetone.top -fmdata FMTRAJECTORY.h5 -fi fminput.dat -mdp mimic.mdp -trr mimic_traj.trr -ndx index.ndx -coords acetone.gro -sele sele.dat \
 -gmx gmx_mpi_d -n_processes 12
```

All the input files can be found in the Zenodo repository associated with this paper (https://doi.org/10.5281/zenodo.17975944).
Figure S1 reports the definition of the different types of atoms, bonds, angles and dihedrals of acetone. Tables S1-S4 report a comparison between a reference parametrization and the parameters obtained using our MiMiCPy-FM protocol. Following the AMBER protocol for non-standard residue parametrization[2], the atomic charges for the reference parametrization were RESP charges obtained

by fitting them to the electrostatic potential of the acetone molecule in vacuum calculated at the B3LYP/6-31G* DFT level using the Gaussian09 program[3]. All remaining force-field parameters were taken from the GAFF force field[4]. As can be seen, the main difference is in electrostatics (Table S1). In particular, we observe an increase of the net charge of oxygen, owing to solvent polarization effects. The bonded parameters remain basically unchanged (Table S2-S4).

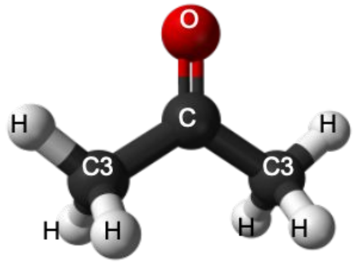

Figure S1: Schematic representation of the acetone molecule showing the atom type assignments (H, C3, C, O), bond types (C3-H, C3-C, C-O), angle types (H-C3-C, C3-C-C3, H-C3-H, C3-C-O), and dihedral types (H-C3-C-O, C3-C3-C-O) used in the force matching procedure. The schematics is used to define the force field parameters reported in Tables S1-S4.

Classical simulations using FM parameters

We used the MiMiCPy-FM parameters to perform classical MD simulations in GROMACS[5]. The equations of motion were integrated with a time step of 2 fs. Temperature was controlled using the velocity-rescaling thermostat (Bussi-Donadio-Parrinello[6]) with a time constant of 0.1 ps, while pressure was maintained at 1.0 bar with the Berendsen barostat[7] using a time constant of 2.0 ps. Long-range electrostatic interactions were treated with the Particle Mesh Ewald (PME) method, using a real-space cutoff of 1.2 nm, while van der Waals interactions were truncated at 1.2 nm using a force-switch modifier starting at 1.0 nm. The following protocol was used: a single acetone molecule was solvated in a box of side 2.5 nm using TIP3P water molecules; the geometry was optimized using the steepest descent algorithm with a force threshold of 100.0 kJ/mol/nm; the system was equilibrated in the NVT ensemble for 10 ns at 300 K; (iv) a 500 ns NPT simulation was performed using the same setup. Three independent NPT production runs have been performed following this protocol. All replicas lead to quantitatively similar results.

In Figs. S2-S4, we compare the distributions of the acetone bond lengths, angles and dihedrals obtained in the reference QM/MM MD simulations, in FM force field-based MD, and in GAFF-based MD simulations. Overall, both the GAFF and FM parameterizations reproduce the reference QM/MM MD results reasonably well. Notably, the FM parameterization is able to better reproduce the QM/MM C-O bond length distribution (Fig. S2i). In Fig. S5, we report the oxygen-oxygen and oxygen-hydrogen radial distribution functions between water molecules and the carbonyl oxygen of acetone. As it can be seen, both GAFF and FM parameterization reproduce the QM/MM OH RDF quite well (Fig. S5a). The FM parameterization leads to a more pronounced dip at 0.3 nm, following the main peak in the OO RDF, accompanied by an increase in probability at ~0.45 nm. This is likely due to the larger electrostatic repulsion following the increase of the carbonyl oxygen point charge when going from GAFF to FM parameterization (see last row in Table S1).

Atom type	MiMiCPy-FM	GAFF
**H**	0.134763	0.1216
**C3**	-0.430329	-0.4767
**C**	0.831756	0.8102
**O**	-0.779672	-0.5864

Table S1: Point charges. The D-RESP charges (units of *e*) obtained from MiMiCPy-FM and the point charges of the GAFF parameterization in vacuum.

	**MiMiCPy-FM**		**GAFF**	
**Bond**	K	R_0	K	R_0
**C3-H**	2.80887E+05	1.09924E-01	2.8225E+05	1.0920E-01
**C3-C**	2.70480E+05	1.51277E-01	2.7472E+05	1.5080E-01
**C-O**	5.41315E+05	1.24656E-01	5.4225E+05	1.2140E-01

Table S2: Bond parameters. Spring constant K ($KJmol^{-1}nm^{-2}$) and equilibrium angle R_0 (nm) – as defined in equation (S1) – obtained from MiMiCPy-FM, and those from the GAFF parameterization in vacuum.

	**MiMiCPy-FM**		**GAFF**	
**Angle**	K	θ_0	K	θ_0
**H-C3-C**	3.92112E+02	108.312	3.9497E+02	109.68
**C3-C-C3**	5.24716E+02	117.954	5.2551E+02	116.05

**H-C3-H**	3.26975E+02	106.342	3.2970E+02	108.35
**C3-C-O**	5.67911E+02	122.200	5.6902E+02	123.11

Table S3: Angle parameters. Spring constant K ($KJmol^{-1}rad^{-2}$) and equilibrium angle θ_0 (deg) – as defined in equation (S2) – obtained from MiMiCPy-FM, and those from the GAFF parameterization in vacuum.

	MiMiCPy-FM						GAFF					
**Dihedral**	**C_0**	**C_1**	**C_2**	**C_3**	**C_4**	**C_5**	**C_0**	**C_1**	**C_2**	**C_3**	**C_4**	**C_5**
**H-C3-C-O**	3.68192	-4.35327	0.0	1.3388	0.0	0.0	3.68192	-4.35136	0.0	1.3388	0.0	0.0
**C3-C3-C-O**	87.864	0.0	-88.0237	0.0	0.0	0.0	87.864	0.0	-87.864	0.0	0.0	0.0

Table S4: Dihedral parameters. The Ryckaert-Bellemans coefficients $C_{0\text{-}5}$ ($KJmol^{-1}$) – defined in equation (S3) – obtained from MiMiCPy-FM, and those from the GAFF parameterization in vacuum.

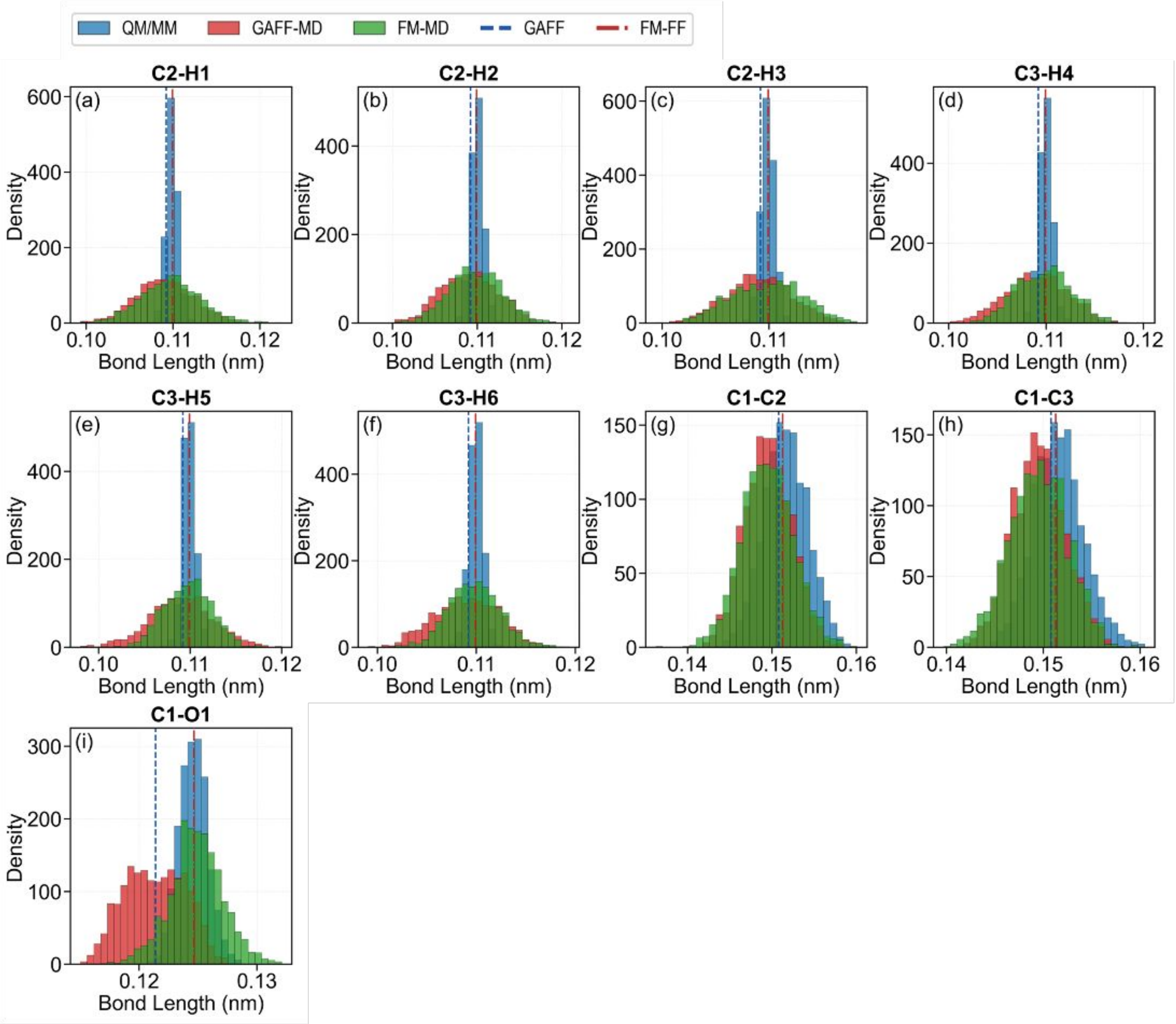

Figure S2: Distribution of covalent bond lengths of acetone obtained from MiMiC QM/MM MD simulations (blue), FM force field MD (green), and GAFF MD (red). Vertical lines indicate average values. Results were obtained from trajectories of equal duration, from which an identical number of samples were extracted, ensuring the same statistical basis across the three different simulations.

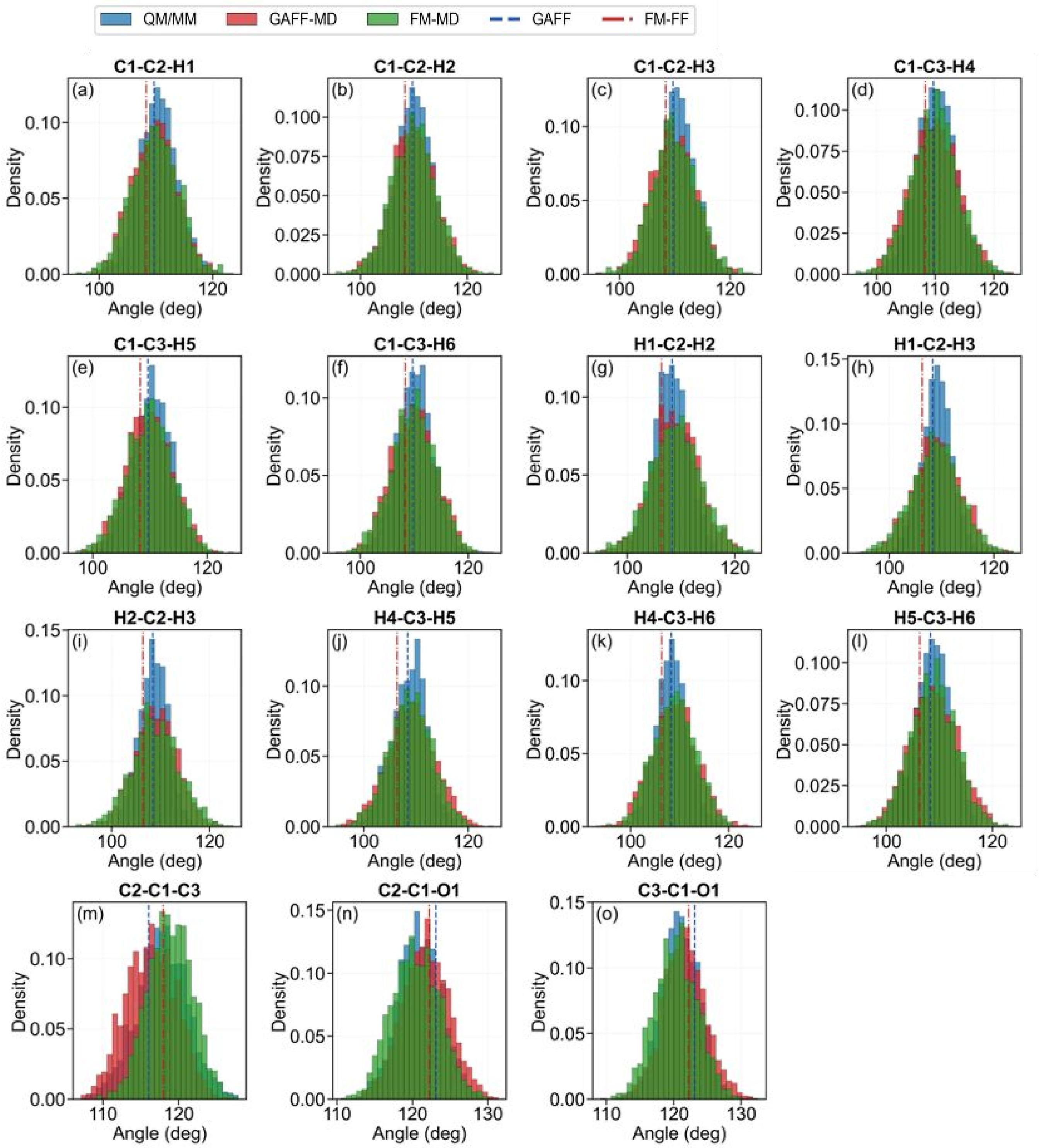

Figure S3: Distribution of angles of acetone obtained from MiMiC QM/MM MD simulations (blue), FM force field MD (green), and GAFF MD (red). Vertical lines indicate average values. Results were obtained from trajectories of equal duration, from which an identical number of samples were extracted, ensuring the same statistical basis across the three different simulations.

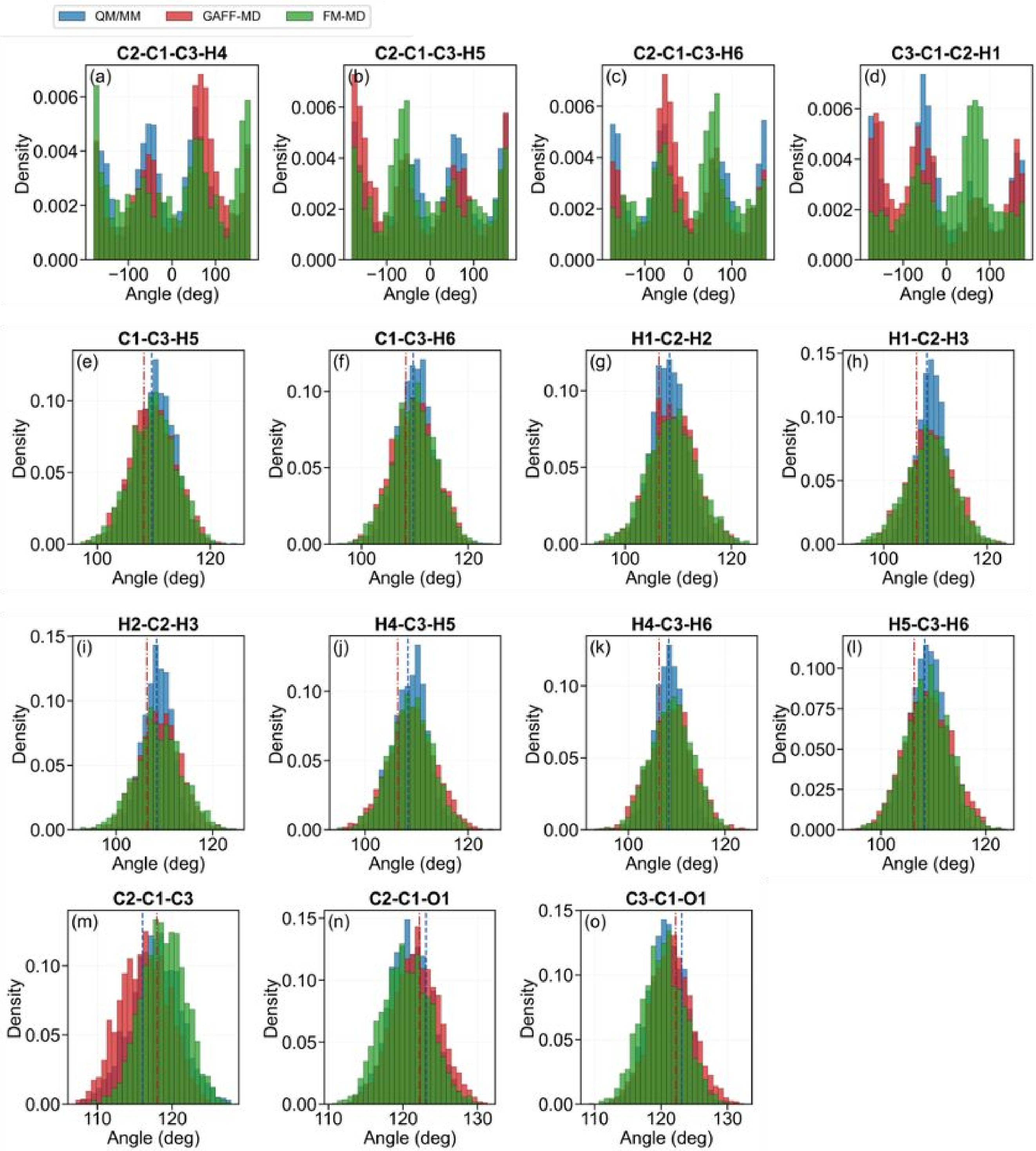

Figure S4: Distribution of dihedrals of acetone obtained from MiMiC QM/MM MD simulations (blue), FM force field MD (green), and GAFF MD (red). Vertical lines indicate average values. Results were obtained from trajectories of equal duration, from which an identical number of samples were extracted, ensuring the same statistical basis across the three different simulations.

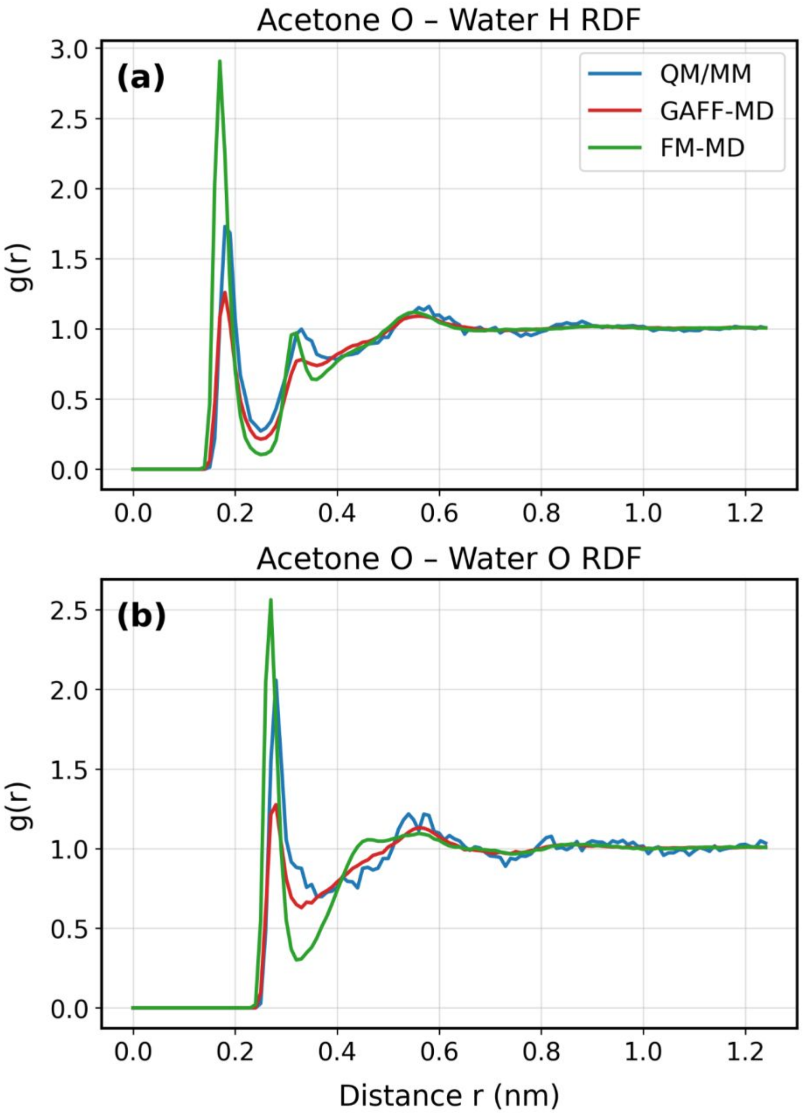

Figure S5: Radial distribution functions (RDFs) characterizing solvation structure around the carbonyl oxygen of acetone in aqueous solution. (a) RDF between the acetone oxygen and water hydrogen atoms. (b) RDF between the acetone and water oxygen atoms.

5. QM/MM calculations on R132H Isocitrate dehydrogenase 1 (IDH1)

We summarize here the QM/MM calculations performed on R132H IDH1, reported in ref [8]. The starting model was based on the R132H mutant IDH1 homodimeric structure (PDB ID: 3INM), which contains Ca^{2+} ions in the active sites of each subunit, the α-ketoglutarate (αKG) substrate, and the NADPH cofactor[9]. To reproduce physiological conditions, the Ca^{2+} ions were replaced with Mg^{2+} ions, and the missing N- and C-terminal residues (Met1–Lys4 and Ala410–Leu414) were added using the *Modeller* progra[10]. QM/MM simulations were performed with the MiMiC code[87], which interfaced CPMD 4.3[9,109] with GROMACS 2019.4[110]. The QM region included αKG, Mg^{2+}, the nicotinamide ring of NADPH, a water molecule bridging the NADPH ribose and the γ-carboxylate of αKG, and residues surrounding both the α-carboxylate of αKG and Mg^{2+} (Arg100/109, His132, Tyr139, Lys212', Asp252/275/279). Additionally, two water molecules coordinating Mg^{2+} and residues involved in binding the γ-carboxylate of αKG (Thr77/75/214', Ser94) were included. The remaining atoms were described at the MM level using the Amber99sb*-ildn force field[14], the TIP3P water mode[15], and literature parameters for NADP[16]. The QM/MM MD protocol consisted of three main stages: minimization, equilibration, and production. During the minimization stage, MM hydrogen atoms were first minimized using the steepest descent algorithm. Subsequently, MM water molecules and sodium ions were minimized for 100 ps while gradually heating the system from 0 to 300 K. Finally, a simulated annealing step was performed to cool the system back to 0 K. The active sites of the two subunits were alternately treated at the QM level, while the Mg^{2+} coordination sphere (αKG, Asp252/275/279, and the two coordinating water molecules) in the opposite subunit was kept constrained. During the equilibration stage, the system was heated to 300 K, again alternating QM treatment between the two active sites while constraining the other. After heating, NVT and NPT simulations were performed, initially applying position restraints to all non-hydrogen atoms, followed by an additional 500 ns of NPT simulation without restraints. Distance restraints were maintained using *PLUMED* version 2.8[17,18], preserving the αKG–Mg^{2+} coordination bond as well as interactions between the bridging water molecule, the NADPH ribose, and the γ-carboxylate of αKG.

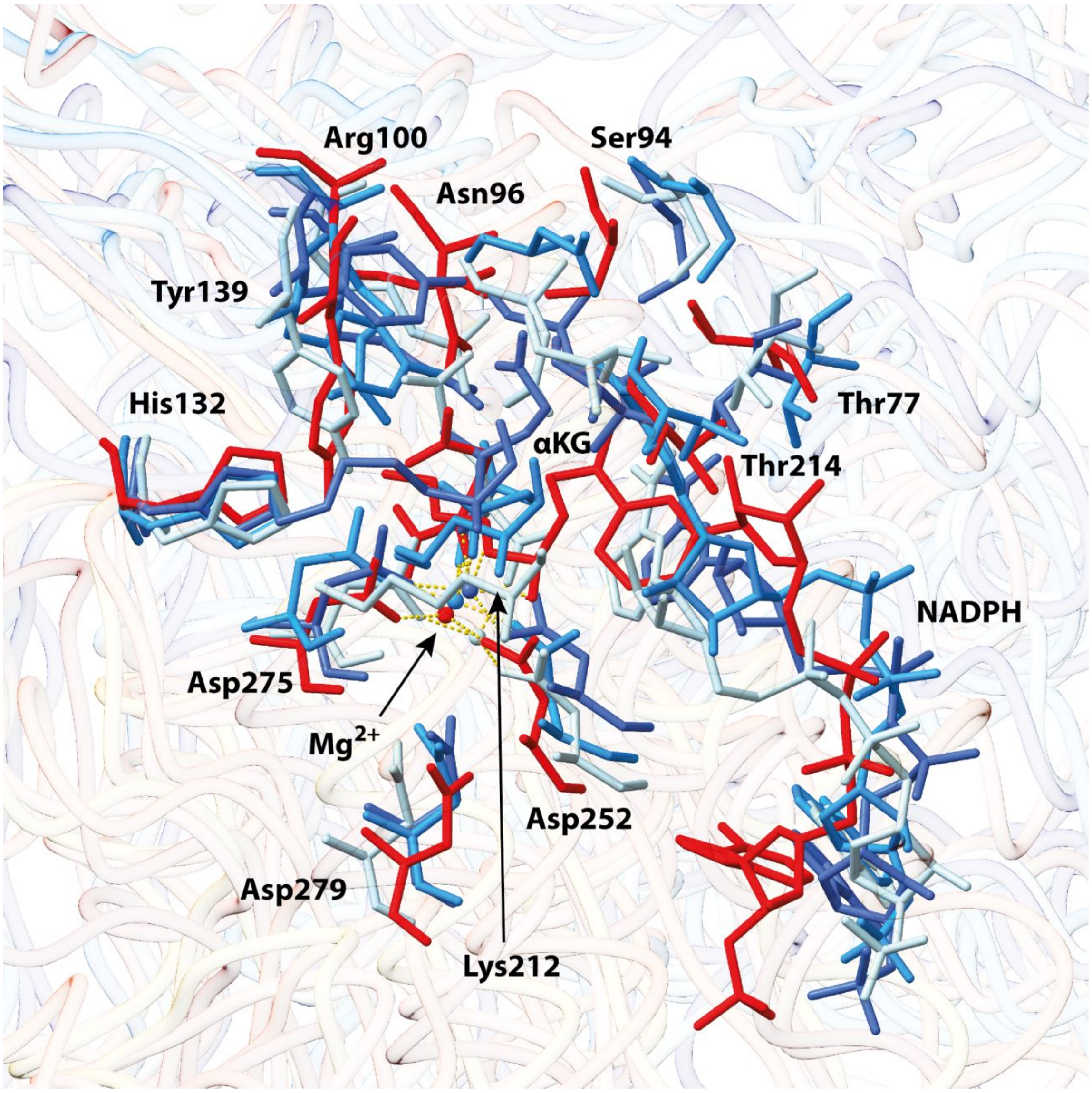

Figure S6. Overlap among the active sites of the three replicas (from dark blue to light blue going from R#1 to R#3) at the end of the dynamics and of the last QM/MM MD snapshot of ref. [8] (in red). The entire QM region of ref. [8] is shown, without hydrogen atoms. Water molecules have been hidden for clarity.

References:

(1) Doemer, M.; Maurer, P.; Campomanes, P.; Tavernelli, I.; Rothlisberger, U. Generalized QM/MM Force Matching Approach Applied to the 11-Cis Protonated Schiff Base Chromophore of Rhodopsin. *J. Chem. Theory Comput.* **2014**, *10* (1), 412–422. https://doi.org/10.1021/ct400697n.

(2) Case, D. A.; Aktulga, H. M.; Belfon, K.; Cerutti, D. S.; Cisneros, G. A.; Cruzeiro, V. W. D.; Forouzesh, N.; Giese, T. J.; Götz, A. W.; Gohlke, H.; Izadi, S.; Kasavajhala, K.; Kaymak, M. C.; King, E.; Kurtzman, T.; Lee, T.-S.; Li, P.; Liu, J.; Luchko, T.; Luo, R.; Manathunga, M.; Machado, M. R.; Nguyen, H. M.; O'Hearn, K. A.; Onufriev, A. V.; Pan, F.; Pantano, S.; Qi, R.; Rahnamoun, A.; Risheh, A.; Schott-Verdugo, S.; Shajan, A.; Swails, J.; Wang, J.; Wei, H.; Wu, X.; Wu, Y.; Zhang, S.; Zhao, S.; Zhu, Q.; Cheatham, T. E.; Roe, D. R.; Roitberg, A.; Simmerling, C.; York, D. M.; Nagan, M. C.; Merz, K. M. AmberTools. *J. Chem. Inf. Model.* **2023**, *63* (20), 6183–6191. https://doi.org/10.1021/acs.jcim.3c01153.

(3) *Gaussian 09, Revision A.02 – ScienceOpen*. https://www.scienceopen.com/document?vid=6be7271f-f651-464b-aee6-ef20b0743b6b (accessed 2025-12-02).

(4) Wang, J.; Wolf, R. M.; Caldwell, J. W.; Kollman, P. A.; Case, D. A. Development and Testing of a General Amber Force Field. *J. Comput. Chem.* **2004**, *25* (9), 1157–1174. https://doi.org/10.1002/jcc.20035.

(5) Abraham, M. J.; Murtola, T.; Schulz, R.; Páll, S.; Smith, J. C.; Hess, B.; Lindahl, E. GROMACS: High Performance Molecular Simulations through Multi-Level Parallelism from Laptops to Supercomputers. *SoftwareX* **2015**, *1–2*, 19–25. https://doi.org/10.1016/j.softx.2015.06.001.

(6) Bussi, G.; Donadio, D.; Parrinello, M. Canonical Sampling through Velocity Rescaling. *J. Chem. Phys.* **2007**, *126* (1), 014101. https://doi.org/10.1063/1.2408420.

(7) Berendsen, H. J. C.; Postma, J. P. M.; van Gunsteren, W. F.; DiNola, A.; Haak, J. R. Molecular Dynamics with Coupling to an External Bath. *J Chem Phys* **1998**, *81*, 3684. https://doi.org/10.1063/1.448118.

(8) Raghavan, B.; De Vivo, M.; Carloni, P. Metal Coordination and Enzymatic Reaction of the Glioma-Target R132H Isocitrate Dehydrogenase 1: Insights by Molecular Simulations. *PLOS One* **2025**, *20* (6), e0326425. https://doi.org/10.1371/journal.pone.0326425.

(9) Dang, L.; White, D. W.; Gross, S.; Bennett, B. D.; Bittinger, M. A.; Driggers, E. M.; Fantin, V. R.; Jang, H. G.; Jin, S.; Keenan, M. C.; Marks, K. M.; Prins, R. M.; Ward, P. S.; Yen, K. E.; Liau, L. M.; Rabinowitz, J. D.; Cantley, L. C.; Thompson, C. B.; Vander Heiden, M. G.; Su, S. M. Cancer-Associated IDH1 Mutations Produce 2-Hydroxyglutarate. *Nature* **2009**, *462* (7274), 739–744. https://doi.org/10.1038/nature08617.

(10) Sali, A.; Blundell, T. L. Comparative Protein Modelling by Satisfaction of Spatial Restraints. *J. Mol. Biol.* **1993**, *234* (3), 779–815. https://doi.org/10.1006/jmbi.1993.1626.

(11) Olsen, J. M. H.; Bolnykh, V.; Meloni, S.; Ippoliti, E.; Bircher, M. P.; Carloni, P.; Rothlisberger, U. MiMiC: A Novel Framework for Multiscale Modeling in Computational Chemistry. *J. Chem. Theory Comput.* **2019**, *15* (6), 3810–3823. https://doi.org/10.1021/acs.jctc.9b00093.

(12) Laio, A.; VandeVondele, J.; Rothlisberger, U. A Hamiltonian Electrostatic Coupling Scheme for Hybrid Car–Parrinello Molecular Dynamics Simulations. *J. Chem. Phys.* **2002**, *116* (16), 6941–6947. https://doi.org/10.1063/1.1462041.

(13) *Car-Parrinello Molecular Dynamics*. GitHub. https://github.com/CPMD-code (accessed 2025-07-24).

(14) Hornak, V.; Abel, R.; Okur, A.; Strockbine, B.; Roitberg, A.; Simmerling, C. Comparison of Multiple Amber Force Fields and Development of Improved Protein Backbone Parameters. *Proteins Struct. Funct. Bioinforma.* **2006**, *65* (3), 712–725. https://doi.org/10.1002/prot.21123.

(15) Mark, P.; Nilsson, L. Structure and Dynamics of the TIP3P, SPC, and SPC/E Water Models at 298 K. *J. Phys. Chem. A* **2001**, *105* (43), 9954–9960. https://doi.org/10.1021/jp003020w.

(16) Holmberg, N.; Ryde, U.; Bülow, L. Redesign of the Coenzyme Specificity in L-Lactate Dehydrogenase from Bacillus Stearothermophilus Using Site-Directed Mutagenesis and Media Engineering. *Protein Eng.* **1999**, *12* (10), 851–856. https://doi.org/10.1093/protein/12.10.851.
(17) *Promoting transparency and reproducibility in enhanced molecular simulations* | *Nature Methods*. https://www.nature.com/articles/s41592-019-0506-8 (accessed 2025-07-24).
(18) Tribello, G. A.; Bonomi, M.; Branduardi, D.; Camilloni, C.; Bussi, G. PLUMED 2: New Feathers for an Old Bird. *Comput. Phys. Commun.* **2014**, *185* (2), 604–613. https://doi.org/10.1016/j.cpc.2013.09.018.
